# Identification of *TRIM14* as a Type I IFN-Stimulated Gene Controlling Hepatitis B Virus Replication by Targeting HBx

**DOI:** 10.3389/fimmu.2018.01872

**Published:** 2018-08-13

**Authors:** Guangyun Tan, Fengchao Xu, Hongxiao Song, Ye Yuan, Qingfei Xiao, Feng Ma, F. Xiao-Feng Qin, Genhong Cheng

**Affiliations:** ^1^Department of Immunology, Institute of Translational Medicine, The First Hospital of Jilin University, Changchun, China; ^2^Department of Medicine Laboratory, The First Hospital of Jilin University, Changchun, China; ^3^Department of Nephrology, The First Hospital, Jilin University, Changchun, China; ^4^Suzhou Institute of Systems Medicine, Suzhou, China; ^5^Department of Microbiology, Immunology and Molecular Genetics, University of California, Los Angeles, CA, United States

**Keywords:** type I IFN, STAT1, TRIM14, hepatitis B virus X protein, hepatitis B virus replication

## Abstract

Hepatitis B virus (HBV) remains a major cause of hepatic disease that threatens human health worldwide. Type I IFN (IFN-I) therapy is an important therapeutic option for HBV patients. The antiviral effect of IFN is mainly mediated *via* upregulation of the expressions of the downstream IFN-stimulated genes. However, the mechanisms by which IFN induces ISG production and inhibits HBV replication are yet to be clarified. TRIM14 was recently reported as a key molecule in the IFN-signaling pathway that regulates IFN production in response to viral infection. In this study, we sought to understand the mechanisms by which IFN restricts HBV replication. We confirmed that TRIM14 is an ISG in the hepatic cells, and that the pattern-recognition receptor ligands polyI:C and polydAdT induce TRIM14 dependent on IFN-I production. In addition, IFN-I-activated STAT1 (but not STAT3) directly bound to the TRIM14 promoter and mediated the induction of TRIM14. Interestingly, TRIM14 played an important role in IFN-I-mediated inhibition of HBV, and the TRIM14 SPRY domain interacted with the C-terminal of HBx, which might block the role of HBx in facilitating HBV replication by inhibiting the formation of the Smc-HBx–DDB1 complex. Thus, our study clearly demonstrates that TRIM14 is a STAT1-dependent ISG, and that the IFN-I–TRIM14–HBx axis shows an alternative way to understand the mechanism by which IFN-I inhibits virus replication.

## Highlights

- The induction of *TRIM14* by IFN-I is STAT1-dependent, but STAT3-independent.
- STAT1 directly binds to the TRIM14 promoter.
- The TRIM14 SPRY domain interacts with the C-terminal of HBx and inhibits the formation of Smc-HBx–DDB1 complex.

## Introduction

The activation of members of the type I interferon (IFN) family (i.e., IFN-α, -β, -ε, -κ, and -ω)—one of the earliest transcriptional responses—is perhaps the most important innate immune response to viral infection. Most nucleated vertebrate cells are capable of both producing and responding to type I IFN (IFN-I). The antiviral function of IFN-I is effected *via* its binding to the IFN-I receptor (IFNAR1), activation of the Janus kinase (JAK)/STAT pathway, and subsequent induction of approximately 300 IFN-stimulated genes (ISGs) (1), which can inhibit different stages of viral life cycle (2, 3). Currently, IFN-I is approved for the treatment of chronic hepatitis B virus (HBV) infection (4), and IFN-α has been shown to inhibit HBV replication *in vitro* (5, 6). However, these treatment strategies are associated with poor response rates and substantial side effects, which impact their clinical utility. Many reports have shown that HBV infection inhibits the production of IFN-I (7, 8), which suggests an antagonistic action of HBV to IFN treatment. Therefore, unraveling the molecular mechanisms of the interaction between IFN and HBV during the course of viral infection is a key imperative that may help improve the efficacy of IFN therapy.

Hepatitis B virus is a partially double-stranded DNA virus that belongs to the *Hepadnaviridae* family (9). On entry into host cells, the viral genome translocates to the nucleus and is converted to covalently closed circular DNA (cccDNA), which is the transcription template for all HBV viral RNAs. The HBV-encoded regulatory protein—hepatitis B virus X (HBx) protein—stimulates HBV gene expression from the cccDNA template. HBx is a 154-amino acid protein with an N-terminal negative regulatory domain and a C-terminal transactivation/coactivation domain; it interacts with several cellular proteins, and its role in viral replication may be mediated through these interactions. The interaction between HBx and damage-specific DNA-binding protein 1 (DDB1) is conserved among HBx proteins from all mammalian *hepadnaviruses* and the woodchuck HBx protein (10). This binding is essential for HBV replication (11). Smc5/6 form the foundation of a multi-subunit DNA-repair complex, and function as a restriction factor by causing selective blockade of extrachromosomal DNA transcription (12). A recent report indicated that the HBx-DDB1-CUL4-ROC1 E3 ligase complex targets the SMC5/6 complex to enhance HBV gene expression from episomal cccDNA (13–15).

Members of the TRIM family are well known for their ring finger E3-ubiquitin ligase activity—comprising a RING domain, 1 or 2 B-box domains, and an associated coiled-coil domain in the amino-terminal region (16–18). As reported by other researchers and ourselves, most TRIMs are IFN-inducible genes, and play a critical role in controlling viral infection (19, 20). We found that TRIM25 was induced by IFN in an IL-27-dependent manner, and it inhibited HBV replication by amplifying IFN signaling (21). Among TRIMs, TRIM14 has recently been recognized as an important molecule that regulates IFN signaling; it was shown to facilitate the activation of IFN-I signaling by recruiting USP14 to cleave the lysine 48 (K48)-linked ubiquitin chains of the cyclic guanosine monophosphate-adenosine monophosphate synthase (cGAS) at K414, thereby inhibiting p62-mediated autophagic degradation of cGAS (22). Moreover, TRIM14 undergoes Lys-63-linked polyubiquitination at Lys-365 and recruits an NF-κB essential modulator to the MAVS signalosome, which leads to activation of both the IFN-regulatory factor 3 and NF-κB pathways and results in an enhanced innate immune response (23). In addition, TRIM14 is required for RIG-I-mediated innate antiviral immunity through the formation of a WHIP–TRIM14–PPP6C mitochondrial signalosome (24). However, the mechanism by which IFN-1 induces TRIM14 in response to viral infection is not well characterized. Moreover, to the best of our knowledge, the function of TRIM14 and the mechanism by which it regulates HBV has not been reported. This study aims to clarify the mechanisms involved in the induction of TRIM14 and the inhibition of HBV replication by TRIM14.

## Materials and Methods

### Samples

In this study, we enrolled 16 HBV-infected patients who were treated with IFN-α (Table S1 in Supplementary Material). Venous blood sampling was done to collect serum and peripheral blood mononuclear cells (PBMCs) 15 min before or 24, 48, and 96 h after IFN-α treatment. We have only collected samples of 10 patients for the 24 h time-point and 6 patients for the 96 h for some reasons. This study (ClinicalTrials.gov number: NCT01671787) was approved by the Ethics Committee of the First Hospital of Jilin University. Written informed consent was obtained from all subjects prior to their enrollment (25).

### Cell Culture, Plasmids, and Reagents

Cells of the HepG2 and HEK293T cell lines were maintained in Dulbecco’s Modified Eagle’s Medium containing 10% inactivated fetal bovine serum. All cell lines were maintained in penicillin (100 IU/mL) and streptomycin (100 mg/mL) in 5% CO_2_ at 37°C. The expression constructs of TRIM14 and STAT1 were generated by cloning the sequence of the coding region into a VR1012 expression vector. Site-directed mutagenesis of STAT1 was generated by QuikChange PCR (TransGen, Beijing, China). The pHBV1.3 plasmids were provided by Dr. Lishan Su, University of North Carolina. Human interleukin (IL)-27 recombinant protein was obtained from R&D Systems (Minneapolis, MN, USA). Human IFN-α was purchased from Peprotech (Jiangsu, China). STAT1, p-STAT1(Tyr701), STAT3, and p-STAT3(Tyr705) antibodies were purchased from Cell Signaling Technology (Danvers, MA, USA). HBsAg antibody was obtained from Thermo (Shanghai, China), HBc antibody was purchased from Abcam (Shanghai, China), anti-tubulin antibody was obtained from Santa Cruz Biotechnology (Santa Cruz, CA, USA), and anti-GAPDH antibody was obtained from Proteintech. Antibodies against TRIM14 and IFNAR1 were purchased from Abcam (Shanghai, China).

### RNA Extraction and Quantitative Real-Time PCR (qRT-PCR)

Total RNA was extracted from cells using the EasyPure RNA Kit (Transgen, China) according to the manufacturer’s instructions and then converted to first-strand cDNA using TransScript First-Strand cDNA Synthesis SuperMix (Transgen, China). HepG2 cells were transfected with pHBV1.3 plasmids and then the medium was changed 6 h after transfection; 66 h later, supernatant was collected and HBV DNA was isolated according to the manufacturer’s instructions enclosed with the kit (Transgen, China). A housekeeping gene, GAPDH, was used as an internal control for quantitation, and gene expression was quantified as previously described (26). For cccDNA detection, DNA was isolated from whole-cell lysates (Transgen, China). Plasmid-safe ATP-dependent DNase (Epicenter) was used to digest the single-strand region of HBV genome, which allowed enrichment of cccDNA for the subsequent real-time PCR detection. Real-time PCR for cccDNA was performed as described previously (27). Gene-specific primer sequences used for qRT-PCR are shown in Table S2 in Supplementary Material.

### Co-Immunoprecipitation and Western Blotting Analysis

Between 24 and 48 h after transfection of expression plasmids, cells were lysed with 50 mM Tris–HCl, pH 8.0, 150 mM NaCl, and 1% NP-40-containing cocktail inhibitors (Selleck). Cell lysates were immunoprecipitated, and then incubated overnight with ANTI-FLAG^®^ M2 Affinity Gel (Sigma). Immunoblotting was carried out as previously described (21). Briefly, cells were collected and lysed in ice-cold cell lysis buffer (20 mM HEPES, 350 mM NaCl, 20% glycerol, 1% NP40, 1 mM MgCl_2_, 0.5 mM EDTA, 0.1 mM EGTA, and 0.5 mM DTT) for 30 min, with tapping of the tubes every 10 min. The protein concentration was quantified by Coomassie Plus™ protein assay reagent (Thermo Scientific). Band intensities were quantified using the ChemiDoc™ XRS^+^ Molecular Imager software (Bio-Rad). Samples were separated by SDS-PAGE and transferred onto polyvinylidene difluoride membranes. The blots were blocked in Tris-buffered saline (50 mM Tris, 150 mM NaCl) containing 0.1% Tween-20 and 5% skimmed milk, and then probed with the relevant antibodies.

### Immunofluorescence

HepG2 cells were transfected with pHBV1.3 plasmids or together with TRIM14 plasmids (GST-HBx or together with Flag-TRIM14 or Flag-EV plasmids); 48 h later, cells were fixed in acetone-methanol (1:1) at 37°C for 10 min. Subsequently, cells were washed with PBS and blocked with 5% BSA in PBST for 1 h, incubated with mouse anti-HBsAg (Thermo) or Flag and GST antibody at 37°C for 1 h, washed in PBS, and then incubated with goat anti-mouse IgG conjugated with FITC or both Cy3(rabbit) and FITC(mouse) conjugated IgG (Proteintech). Cells were washed with PBS and observed under a fluorescence microscope.

### Enzyme-Linked Immunosorbent Assay

HepG2 cells were mock transfected or transfected with TRIM14-expression plasmids together with pHBV1.3-HBV expression plasmids. The supernatant was collected after 72 h, and the supernatant from HepG2-NTCP cells was collected 9 days after infection to detect the levels of HBV e-antigen (HBeAg) and HBV surface antigen by ELISA (HBsAg; Kehua Biotech, China).

### CRISPR/Cas9 Knockout

HepG2 or HepG2-NTCP cells were seeded on 24-well plate; after 16 h, plasmids expressing Cas9, sgRNA, and a plasmid with a puromycin selection marker were co-transfected into HepG2 cells using Viafect transfection reagent (Promega). At 36 h posttransfection, cells were either selected by adding puromycin (2 µg/mL) or subjected to immunoblotting with TRIM14-specific antibodies. Two days later, serial dilution of living cells was done to obtain a cell density of 1 cell per well on a 96-well plate. Immunoblotting was repeated to ensure gene knockout (KO) results after the clones were grown, and DNA sequencing was undertaken to further confirm the results of gene KO. sgRNA sequences are shown in Table S2 in Supplementary Material.

### TRIM14 Promoter Reporters and Dual-Luciferase Reporter Assay

TRIM14 promoter reporters were generated by cloning the promoter region sequence into a pGL4.11 expression vector. HEK293T cells were transfected with the respective TRIM14 promoter reporters together with a pGL4.74 Tk-Rluc reporter. Sixteen hours later, cells were either treated with IFN-α or transfected with polyI:C or polydAdT; after 24 h of this treatment, cells were lysed with passive lysis buffer (Promega). To detect the relative production of IFN-β, HepG2 cells were first transfected with dual-luciferase construct harboring IFN-β target sequence together with a pGL4.74 Tk-Rluc reporter. After 8 h, the cells were transfected with polyIC, polydAdT, or pHBV1.3 plasmid; 16 h later, the cells were lysed with passive lysis buffer and the activity of Firefly Luciferase and Renilla Luciferase in the lysates was measured with the dual-Luciferase Assay System (Promega, Madison, WI, USA).

### Chromatin Immunoprecipitation (ChIP)

Chromatin immunoprecipitation assays were carried out as described earlier (26). Briefly, the treated cells were cross-linked with 1% formaldehyde, sheared to an average size of ~500 bp, and then immunoprecipitated with immunoglobulin G (IgG) or antibodies against STAT1 or STAT3. The ChIP-PCR primers (Table S2 in Supplementary Material) were designed to amplify the proximal promoter regions containing putative STAT1-binding sites within the TRIM14 promoter.

### HBV Infection Assay

Serum of HBV patients was collected and concentrated. HepG2-NTCP WT or TRIM14 KO cells were pretreated with or without IFNα (10 ng/mL) for 24 h, and were inoculated with HBV for another 24 h with a multiplicity of infection of 100 genome equivalents per cell (4% PEG8000). After infection, cells were washed three times with PBS, and were maintained in DMEM medium for another 9 days; the medium was changed every 2 days. The supernatant and cells were collected for detection of HBV DNA, pre-genomic RNA (pgRNA), HBsAg, and HBeAg by Q-PCR or ELISA. Expression of *TRIM14* was detected by Western Blot.

### Statistical Analysis

Results were presented as mean ± SD, and analyzed by using Student’s *t*-test. *P* < 0.05 was considered as indicative of a statistically significant difference.

## Results

### TRIM14, an ISG, Is Upregulated in HBV-Infected Patients on IFN Treatment

**Figure 1:**
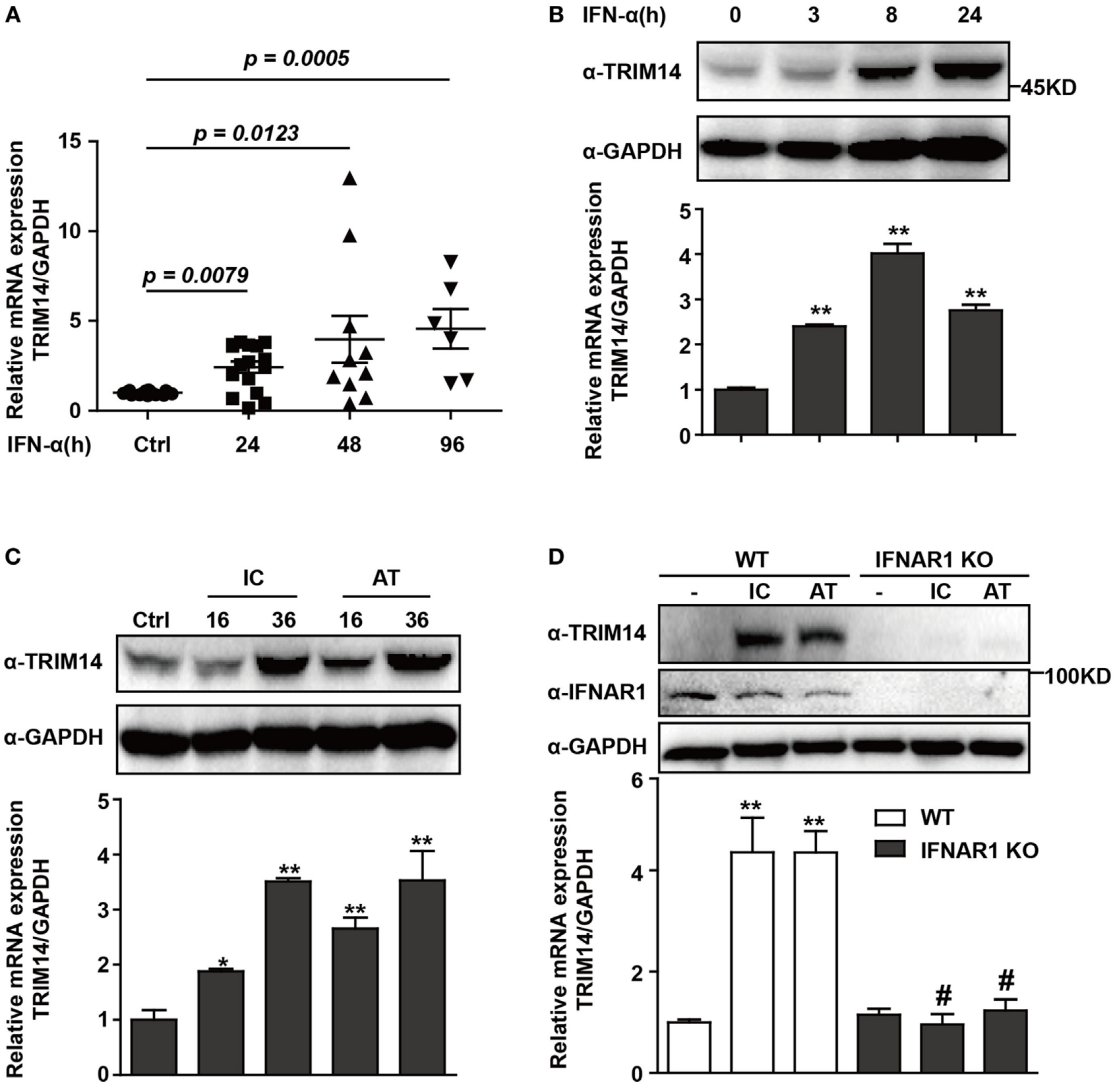
TRIM14 is induced by IFN-I treatment. **(A)** Total RNA was extracted from peripheral blood mononuclear cells isolated from 16 HBV patients, with samples collected before or after IFN-α treatment (Ctrl/16, 24 h/16, 48 h/10, and 96 h/6). The expression level of TRIM14 was analyzed using quantitative real-time PCR (qRT-PCR). **(B)** HepG2 cells were treated with IFN-α (10 ng/mL) as indicated, and whole-cell lysates were immunoblotted with TRIM14 and GAPDH antibodies. RNA was extracted and qRT-PCR was conducted to determine TRIM14 expression. **(C)** HepG2 cells were transfected with polyI:C or polydAdT (1 µg/mL) as indicated, and analyzed as described in **(B)**. **(D)** HepG2 WT or IFNAR1 KO cells were transfected with polyI:C or polydAdT as indicated; cells were collected after 36 h and analyzed as specified in **(B)**. Mean (±SD) values from three independent experiments are presented. Student’s *t-*test was applied to analyze results. *p < 0.05; **p < 0.01, and ^#^*p* > 0.05.

To investigate the expression of TRIM14 in HBV patients on IFN-α treatment, PBMCs from samples collected at different time points before or after IFN-α treatment were isolated from the peripheral blood of HBV patients, and TRIM14 mRNA expression was analyzed by qRT-PCR. As expected, TRIM14 was significantly upregulated in the PBMCs of patients following IFN-α treatment (Figure 1A). As a validation of TRIM14 induction in hepatic cells, we found that IFN-α induced both protein and mRNA expressions of TRIM14 in HepG2 cells (Figure 1B). Previous results from our experiments and those of other researchers showed that polyI:C or polydAdT promotes IFN-I production (Figure S1 in Supplementary Material) (21, 28). In this study, we found HepG2 cells transfected with polyI:C or polydAdT significantly activated TRIM14 expression (Figure 1C). Moreover, we generated a HepG2 cell line with IFNAR1 KO by CRISPR/Cas9 technology; interestingly, the induction of TRIM14 by polyI:C or polydAdT was almost entirely blocked, which confirmed our conjecture that TRIM14 is an ISG that could be induced in hepatic cells (Figure 1D). Collectively, these data demonstrate that TRIM14 is an IFN-I-inducible gene in hepatic cells and might play a role in the anti-HBV response.

### IL-27 Is Not Indispensable for TRIM14 Induction by IFN-I

**Figure 2:**
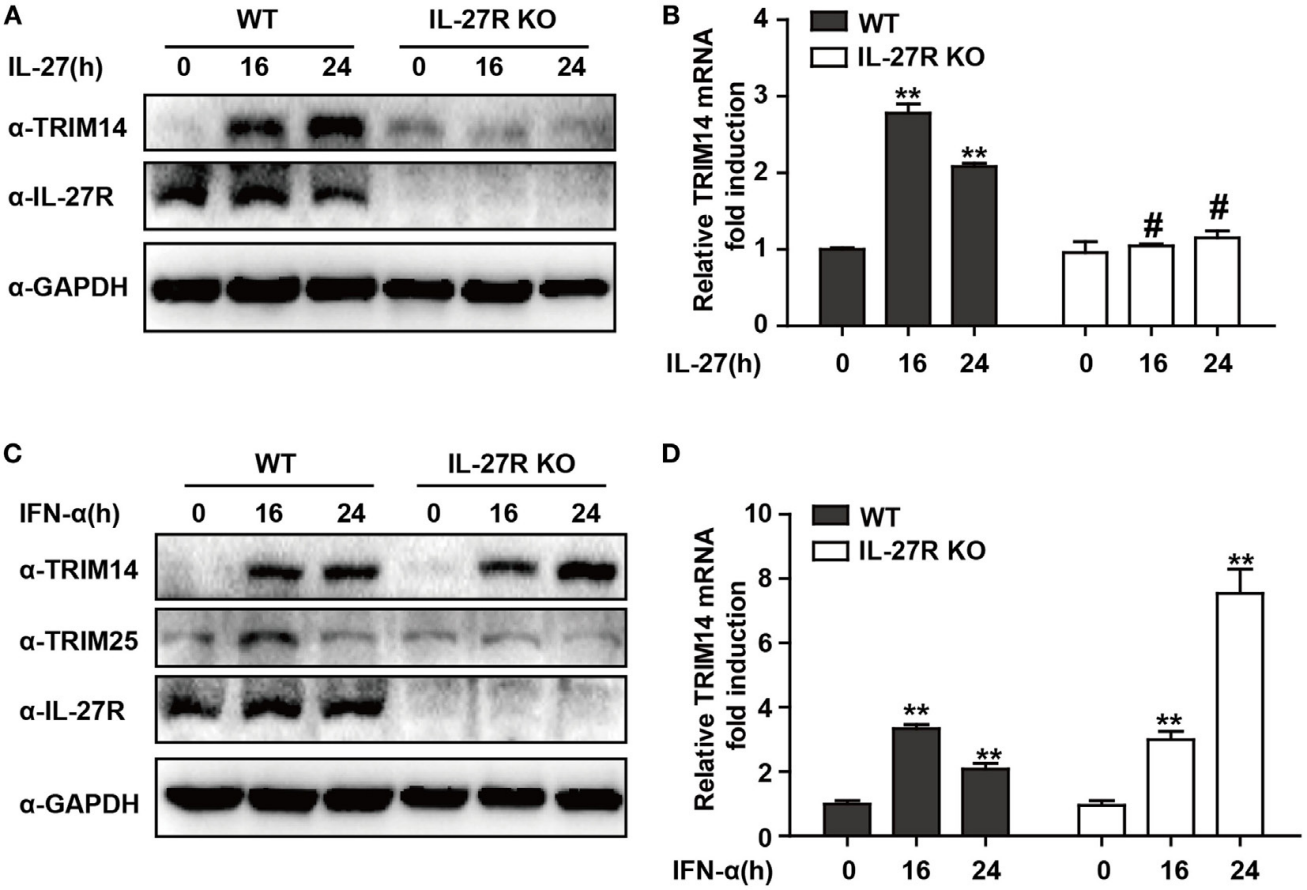
IFN-I-mediated TRIM14 induction is not dependent on IL-27. **(A)** HepG2 WT or IL-27R KO cells were treated with IL-27 (10 ng/mL), and immunoblotted with TRIM14, IL-27R, or GAPDH antibody **(B)** Total RNA was extracted from **(A)** and the TRIM14 expression level was determined using quantitative real-time PCR (qRT-PCR). **(C)** HepG2 WT or IL-27R KO cells were treated with IFN-α (10 ng/mL) as indicated, and immunoblotted with TRIM14, TRIM25, IL-27R, or GAPDH antibody. **(B)** Total RNA was extracted from **(C)**, and **(D)** the TRIM14 expression level was determined using qRT-PCR. Mean (±SD) values from three independent experiments are presented. Student’s *t-*test was applied to analyze results. *p < 0.05; **p < 0.01; and ^#^*p* > 0.05.

IFN-I-induced IL-27 secretion is responsible for activation of both STAT1 and STAT3 (29, 30). In hepatic cells, IL-27 was reported to modulate CXCL9, CXCL10, and CXCL11 chemokines (31). Consistently, in a recent study, we showed that TRIM25—induced by IFN-I in an IL-27-dependent manner—might inhibit HBV replication by promoting IFN production (21). We evaluated whether IL-27 was important in IFN-I-induced TRIM14 expression. As expected, IL-27 protein stimulation increased TRIM14 expression whereas IL-27R KO blocked this process (Figures 2A,B). Following IFN-α treatment, expression of TRIM25 was induced in HepG2 WT cells and blocked in IL-27R KO HepG2 cells. In contrast, however, there was greater TRIM14 induction in IL-27R KO HepG2 cells after IFN-α treatment as compared to that in WT cells (Figures 2C,D). These data indicate that IL-27 is not indispensable in IFN-I-mediated induction of TRIM14.

### STAT1 Is Essential for IFN-I Induction of TRIM14

**Figure 3:**
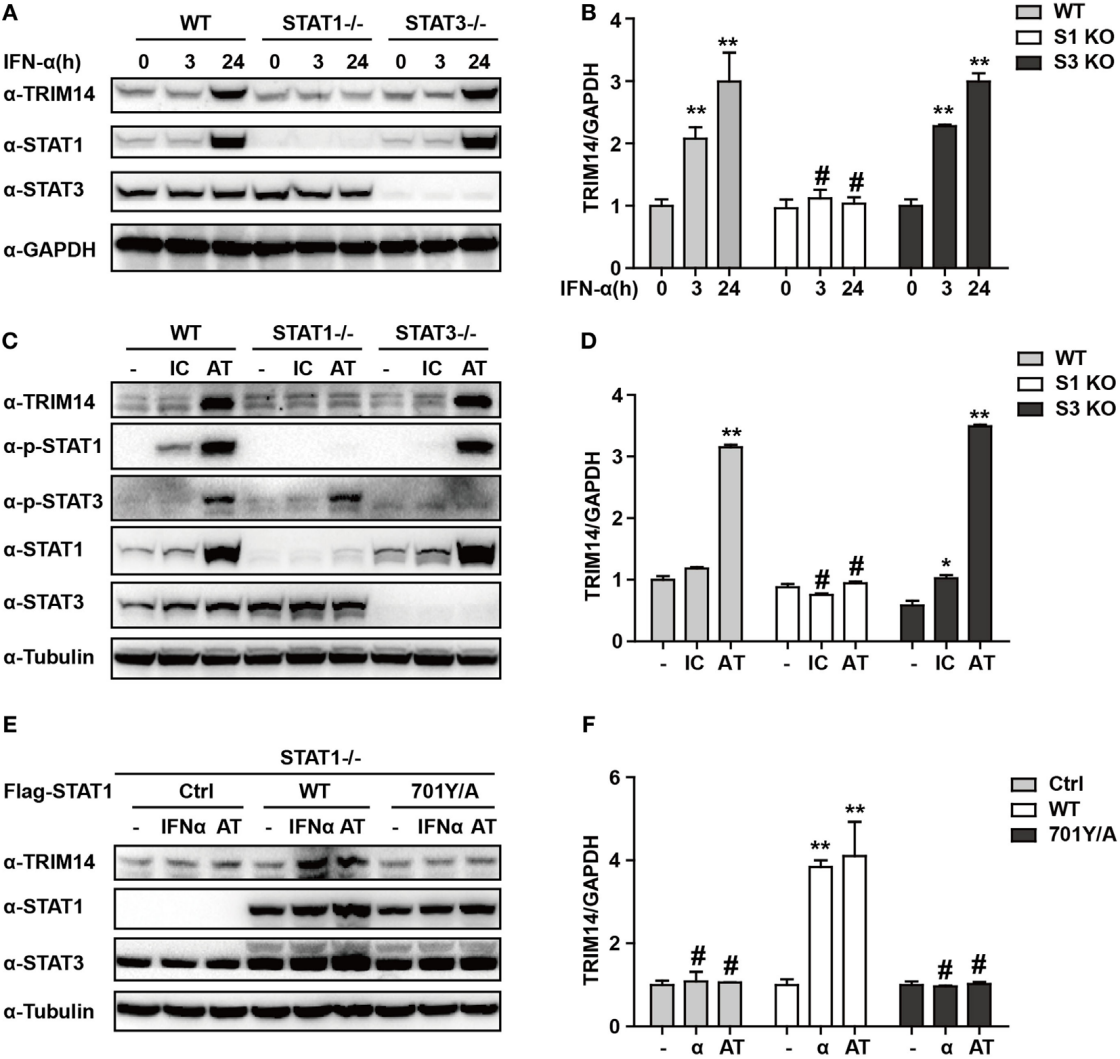
IFN-I induction of TRIM14 is STAT1 dependent. **(A)** WT, STAT1 KO, and STAT3 KO 293 T cells were treated with IFN-α (10 ng/mL) as indicated, and whole-cell lysates were immunoblotted with the indicated antibodies. **(B)** WT, STAT1 KO, and STAT3 KO 293 T cells were treated with IFN-α (10 ng/mL) as indicated; RNA was extracted and subjected to quantitative real-time PCR (qRT-PCR) to determine TRIM14 expression. **(C,D)** WT, STAT1 KO, and STAT3 KO 293 T cells were transfected with polyI:C or polydAdT (1 µg/mL); 24 h later, whole-cell lysates were immunoblotted with the indicated antibodies. RNA was extracted and subjected to qRT-PCR to determine TRIM14 expression. **(E,F)** STAT1 KO 293 T cells were transfected with STAT1 WT, 701Y/A mutation expression plasmids, or empty vector; 24 h later, cells were treated with IFN-α or transfected with polydAdT as indicated; after another 24 h, cells were collected and analyzed as specified in **(A,B)**.

We have shown that TRIM14 is an IL-27-independent ISG. To further verify the mechanisms through which IFN-I induces TRIM14, we hypothesized that IFN-α may induce TRIM14 by directly activating members of the STAT family. Therefore, 293 T cells KO for STAT1 or STAT3 were used. Interestingly, TRIM14 was not induced by IFN-α in STAT1 KO cells, but was upregulated in STAT3 KO cells (Figures 3A,B). In addition, by transfection of polyI:C or polydAdT into cells that would result in IFN-I induction, we confirmed that STAT1, but not STAT3, played a role in TRIM14 induction (Figures 3C,D). In the canonical IFN-signaling pathway, all types of IFNs produce transcriptionally active STAT1 through JAK-mediated phosphorylation at Y701 (32). We further found that TRIM14 induction was restored upon reconstitution of STAT1 in STAT1 KO cells by using a plasmid expressing WT STAT1, but not 701Y/A STAT1 (Figures 3E,F). Taken together, these results demonstrate that the activation of STAT1 is essential for IFN-I induction of TRIM14.

### Identification of STAT1-Binding Motif in TRIM14 Promoter

**Figure 4:**
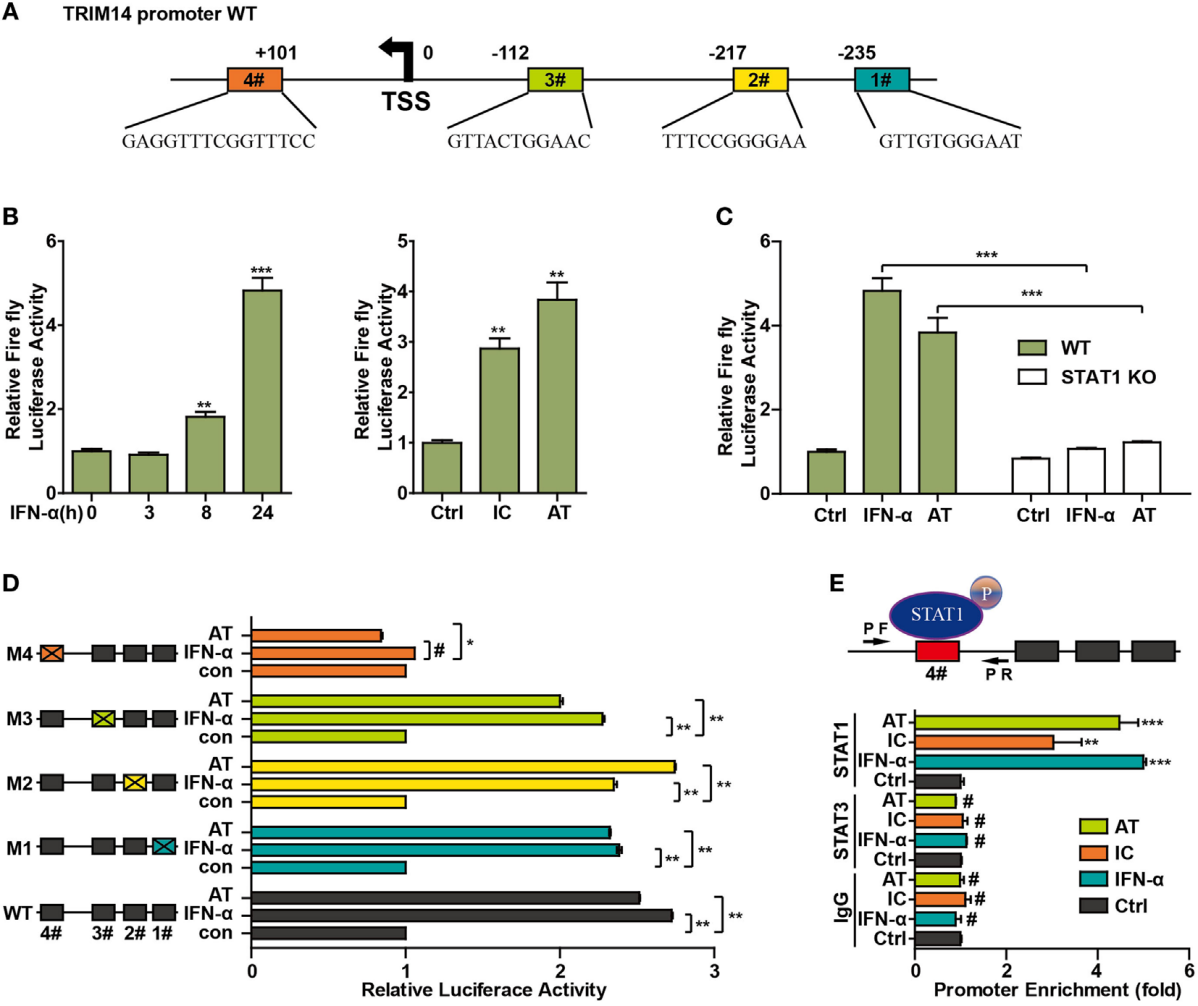
STAT1 binds to the TRIM14 promoter. **(A)** Schematic illustration showing potential STAT1 binding sites in the TRIM14 promoter region. **(B)** 293 T cells were transfected with luciferase reporters harboring wild-type (WT) promoter regions of TRIM14 together with pGL4.74 TK-Luc reporter; 16 h later, cells were treated with IFN-α (10 ng/mL) or transfected with polyI:C or polydAdT; luciferase activity was measured 24 h after treatment. Firefly luciferase activity was first normalized to that of Renilla luciferase, and fold induction of relative luciferase activity over control group was plotted as shown. **(C)** WT or STAT1 KO 293 T cells were treated or transfected as indicated, and analyzed as in **(B)**. **(D)** 293 T cells were transfected with luciferase reporters harboring WT or mutated promoter regions of TRIM14 together with pGL4.7 TK-Luc reporter; 16 h later, cells were treated with IFN-α (10 ng/mL) or transfected with polydAdT, and analyzed as in **(B)**. **(E)** HepG2 cells were treated by IFN-α for 24 h or transfected with polyI:C or polydAdT; 24 h later, a chromatin immunoprecipitation assay was undertaken to analyze STAT1 binding to site (#4) within the TRIM14 promoter region. STAT3 was used as a negative control.

To further investigate the mechanism of TRIM14 induction by IFN-I, the potential transcription factor (TF)-binding sites in the human TRIM14 gene promoter region were predicted by JASPAR (33) and TRANSFAC (34). Conservation analysis of the TF-binding sites among the mammalian species was analyzed and viewed by a UCSC genome browser (http://genome.ucsc.edu/). Four potential STAT1-binding sites were observed in the TRIM14 promoter region (Figure 4A); 820 bp of the promoter, including these four sites, was amplified and cloned into a pGL4.1 luciferase reporter vector to generate WT-luc and Site Deletion-luc reporter constructs. Our results showed that both IFN-α treatment and transfection of polyI:C or polydAdT significantly induced WT-luc activity (Figure 4B), whereas this induction was blocked in the STAT1 KO cells (Figure 4C). This suggests that STAT1 is the activator of TRIM14 gene promoter luciferase activity. To further confirm the binding site, potential sites were deleted individually, as shown in Figure 4D. Interestingly, luciferase reporter assay indicated that only Site #4 was required for TRIM14 transcription upon IFN-α treatment or polydAdT transfection (Figure 4D). Finally, two pairs of ChIP primers (one for #4 and the other for #1 and #2) were designed as indicated (Figure 4E; Figure S2 in Supplementary Material), and a ChIP assay was conducted to verify the direct binding of STAT1 to the TRIM14 promoter. As indicated in Figure 4E, in response to IFN-α treatment or transfection of polyI:C or polydAdT, Q-PCR results showed no signal in the IgG and STAT3 antibody ChIP samples, while significant induction was detected in the STAT1 ChIP sample. This demonstrated that IFN-α treatment or transfection of polyI:C or polydAdT significantly promoted recruitment of STAT1, but not that of STAT3, to TRIM14 gene promoter regions (#4). In addition, we confirmed that STAT1 was not recruited to Site #1 or #2 (Figure S2 in Supplementary Material). Taken together, these results support the critical role of STAT1 in mediating TRIM14 induction upon IFN-α treatment.

### TRIM14 Inhibits HBV Infection and Replication and Is Important for IFN-I-Mediated Inhibition of HBV

**Figure 5:**
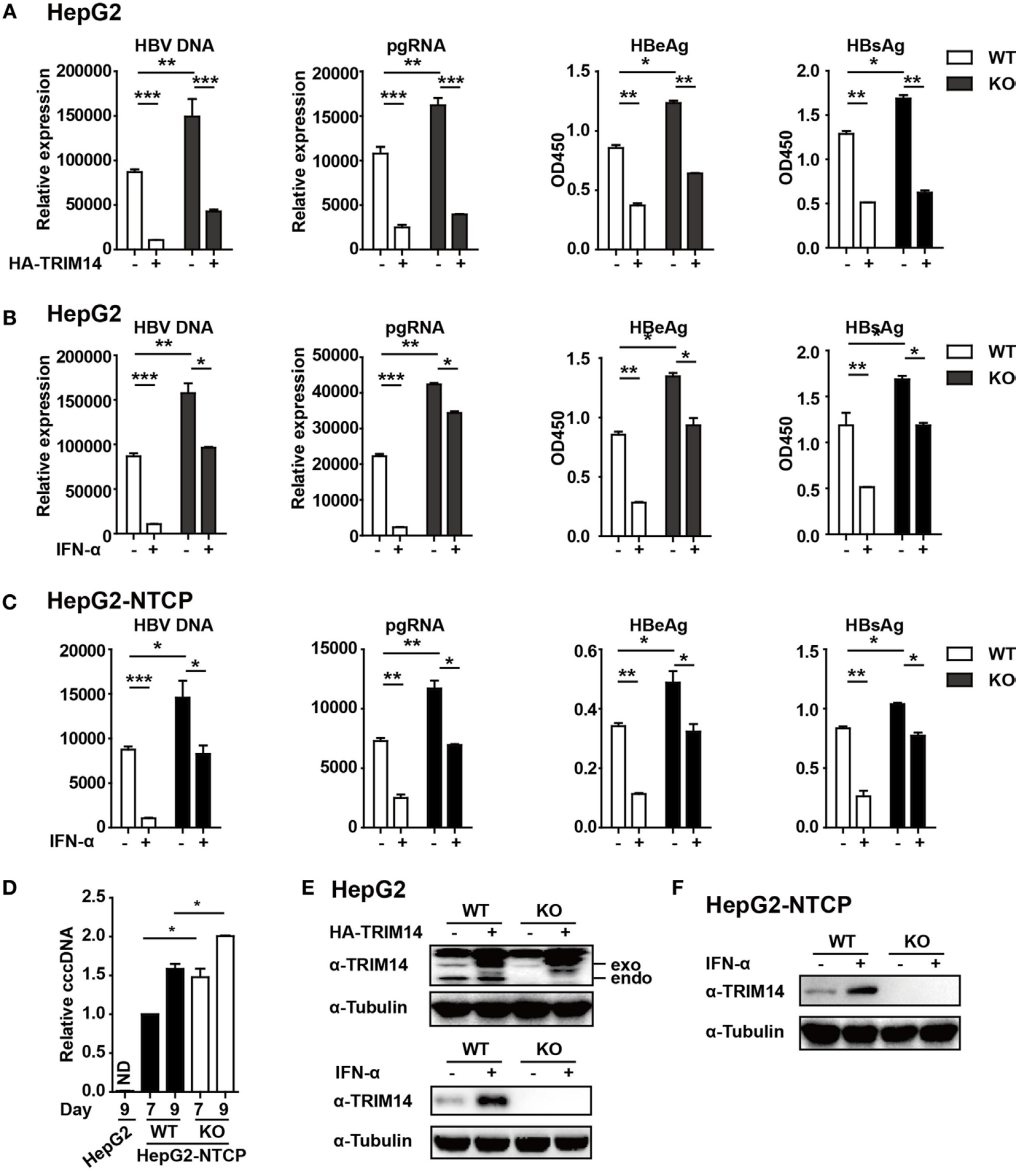
TRIM14 is important for IFN-I-mediated inhibition of hepatitis B virus (HBV). **(A)** HepG2 WT or TRIM14 KO cells were transfected with pHBV1.3 or co-transfected with pHBV 1.3 and TRIM14 as indicated. At 72 h, the supernatant was collected and cells were subjected to immunoblotting with antibodies as indicated. RNA and HBV DNA were isolated from the cells or supernatants and analyzed by quantitative real-time PCR (qRT-PCR). HBeAg and HBsAg in the supernatant were analyzed by ELISA. Mean ± SD values from three pooled independent experiments are presented. *p < 0.05 and **p < 0.01. **(B)** HepG2 WT and TRIM14 KO cells were pretreated with IFN-α for 24 h and transfected with pHBV1.3; 48 h later, the supernatant was collected and cells were subjected to immunoblotting with antibodies as indicated. RNA and HBV DNA were isolated from the cells or supernatants and analyzed by qRT-PCR. HBeAg and HBsAg levels in the supernatant were determined by ELISA. **(C)** HepG2-NTCP WT and TRIM14 KO cells were pretreated with IFN-α for 24 h and infected with HBV; 9 days later, cells and supernatant were collected and analyzed as in **(B)**. **(D)** HepG2-NTCP WT and TRIM14 KO cells were infected with HBV (HepG2 cells as a negative control); 7 or 9 days after infection, covalently closed circular DNA was isolated from the whole cell lysates and quantified by qRT-PCR. **(E,F)** Whole-cell lysates from **(A–C)** were immunoblotted with TRIM14 and GAPDH antibodies. All data are from three pooled independent experiments and shown as mean ± SD. *p < 0.05 and **p < 0.01.

In continuation with the above-mentioned findings, we further investigated the effect of TRIM14 on HBV replication. The pHBV1.3 plasmid can be used to transfect HepG2 cells and create an infectious HBV virus. This plasmid was co-transfected with HA-tagged TRIM14-expressing plasmids to evaluate the possible effects on viral replication. Cells and supernatants were collected at 72 h after transfection. Levels of HBV DNA, HBV pgRNA, HBsAg, and HBeAg were quantified through qRT-PCR and ELISA analysis. Notably, HBV DNA, pgRNA, HBsAg, and HBeAg were significantly reduced after TRIM14 overexpression, and significantly increased in TRIM14 KO HepG2 cells (Figures 5A,E), which suggests an inhibitory effect of TRIM14 on HBV replication. In addition, Western blotting analysis showed that the protein levels of HBs and HBc were reduced by TRIM14; in addition, immunofluorescence analyses revealed that HBs expression was decreased by TRIM14 (Figure S3 in Supplementary Material). To further confirm the role of TRIM14 in IFN-I-mediated HBV inhibition, HepG2 WT or TRIM14 KO cells were pretreated with IFN-α for 24 h and transfected with pHBV1.3 plasmids. We found that the inhibitory effect of IFN-α on HBV replication was significantly attenuated in TRIM14 KO cells (Figures 5B,E). The induction of other classical ISGs (e.g., IFIT2, IFIT3, and IFIT5) was not affected by TRIM14 KO after IFN treatment (Figure S4 in Supplementary Material). In addition, we generated a TRIM14 KO HepG2-NTCP cell line by CRISPR/Cas9 technology; we treated the cells with IFN-α for 24 h and subsequently infected them with HBV. The results (Figures 5C,F) were similar to those shown in Figure 5B. We also tested cccDNA formation in the WT or TRIM14 KO HepG2-NTCP cells; the results indicated that cccDNA was significantly increased after TRIM14 knockout (Figure 5D). These data demonstrate an important role of TRIM14 in IFN-I-mediated inhibition of HBV replication.

### TRIM14 Interacts With the C-Terminal of HBx and Inhibits the Formation of Smc-HBx–DDB1 Complex

**Figure 6:**
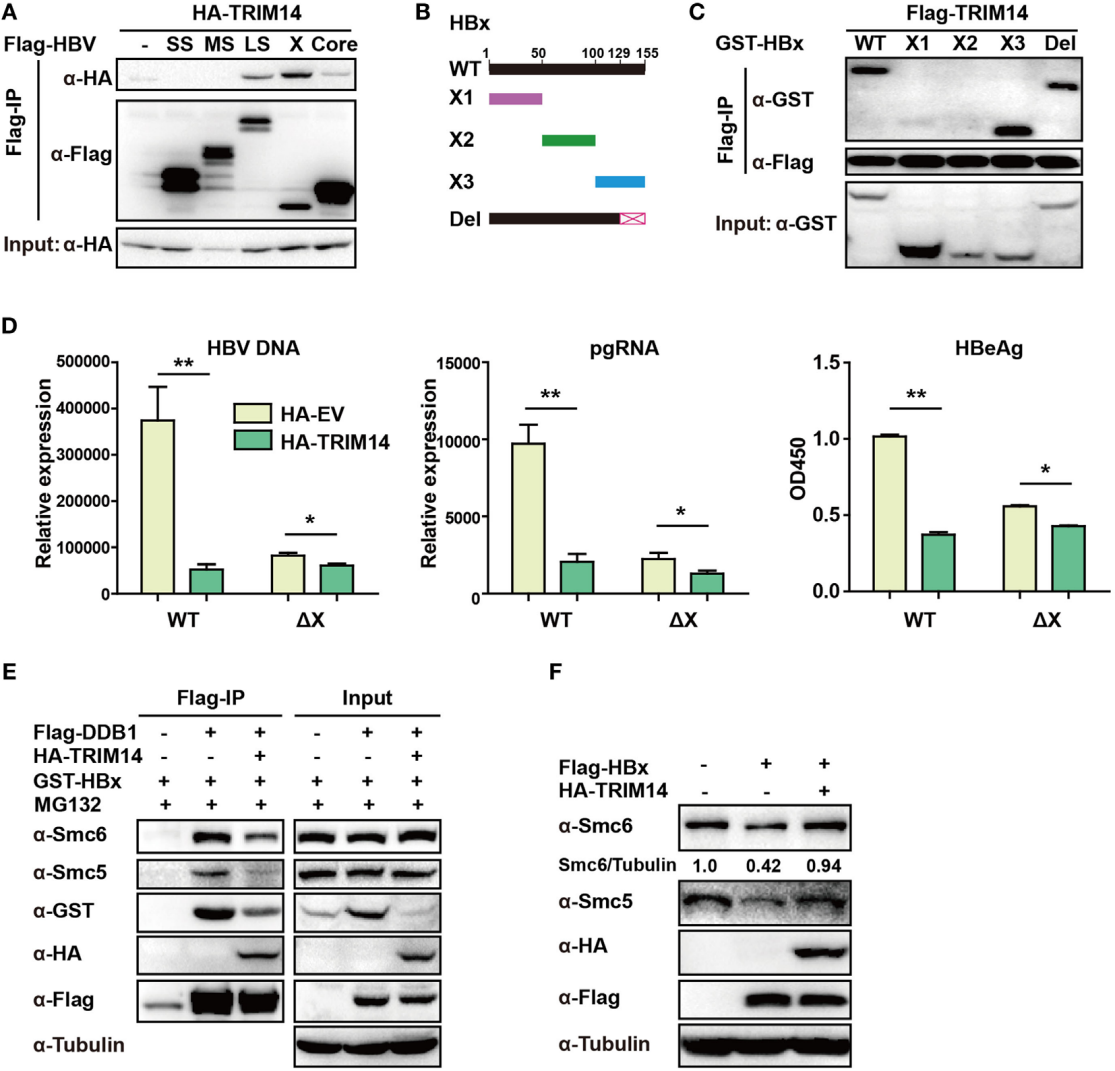
TRIM14 interacts with the C-terminal of hepatitis B virus X (HBx) protein. **(A)** 293 T cells were transfected with HA-TRIM14 or co-transfected with HA-TRIM14 and Flag-tagged HBV protein expression plasmids as indicated; 40 h later, cells were collected and subjected to co-immunoprecipitation assay; the blot was immunoblotted with antibodies as indicated. **(B)** Diagrams of mutant HBx constructs; the numbers indicate the amino acids in the HBx constructs. **(C)** 293 T cells were co-transfected with Flag-TRIM14 and WT GST-HBx protein expression plasmids or mutants as indicated and analyzed as in **(A)**. **(D)** HepG2 cells were transfected with pHBV1.2 WT or pHBV 1.2ΔX plasmids with or without TRIM14, as indicated; at 72 h, RNA and HBV DNA were isolated from the cells or supernatants and analyzed by quantitative real-time PCR (qRT-PCR). HBeAg level in the supernatant was determined by ELISA. Data are presented as mean ± SD from three independent experiments. The student’s *t-*test was used to analyze results. *p < 0.05 and **p < 0.01. **(E)** 293 T cells were transfected with HA-TRIM14, Flag-DDB1, or GST-HBx expression plasmids as indicated. Thirty-six hours later, cells were treated with MG132 (10 μM) for 8 h; cells were collected and subjected to CO-IP assay; the blot was immunoblotted with antibodies as indicated. **(F)** HepG2 cells were transfected with HA-TRIM14 or Flag-HBx expression plasmids as indicated. Forty-eight hours later, cells were collected and subjected to immunoblotting with antibodies as indicated.

TRIM14 was shown to initiate innate immunity by promoting the production of type I IFN (22). In order to verify if the TRIM14-mediated inhibition of HBV replication was type I IFN-dependent, pHBV1.3 plasmid was co-transfected with HA-tagged TRIM14-expressing plasmids into WT or IFNAR1 KO HepG2 cells; we found that HBV replication in IFNAR1 KO HepG2 cells was still inhibited by TRIM14 (Figure S5 in Supplementary Material). The results indicate that TRIM14 may inhibit HBV replication, at least partially, in an IFN-I-independent manner. Protein–protein interaction is perhaps the most direct pattern seen in the gene biologic function (35). Based on this premise, we investigated whether TRIM14 could interact with HBV proteins. Five HBV proteins—HBV core, small S, middle S, large S, and X—were co-transfected individually with TRIM14 expression plasmids into 293 T cells. Results suggested that TRIM14 interacted with HBx, rather than with the other HBV proteins (Figure 6A), and the interaction of TRIM14 and HBx was confirmed by immunofluorescence analysis (Figure S6 in Supplementary Material). Given that HBx mediates viral replication through cellular protein interactions, we further created four HBx mutants (Figure 6B) to identify the interaction region of TRIM14 and HBx. As surmised, TRIM14 bound to the 100–128 amino acid region located at the C-terminal of HBx (Figure 6C), which suggests that the interaction might inhibit the role of HBx in HBV replication. To verify whether TRIM14 inhibited HBV replication through HBx, pHBV1.2 WT or ΔX (without HBx expression) was cotransfected with TRIM14 or empty vector into HepG2 cells; 72 h later, ELISA and qRT-PCR were performed to quantify protein levels of HBeAg, HBV DNA, and HBV pgRNA. The results indicated that HBV replication was largely inhibited in pHBV1.2ΔX-transfected cells as compared to that in pHBV1.2WT-transfected cells; these findings suggest an important role of HBx in promoting HBV replication. Furthermore, the effect of TRIM14 inhibition on HBV replication in pHBV1.2ΔX-transfected HepG2 cells was much less significant than that in pHBV1.2 WT-transfected cells (Figure 6D). This demonstrates that TRIM14 inhibited HBV replication at least partially by targeting HBx. In addition, we sought to determine whether the binding of TRIM14 has any impact on the function of HBx. Interestingly, as shown in Figure 6E, HBx and Smc5/6 significantly interacted with DDB1 in the CoIP assay; however, after co-transfection with TRIM14 plasmids, the interaction was almost blocked, which indicated that TRIM14 inhibited the formation of Smc-HBx–DDB1 complex. In addition, we found that TRIM14 significantly inhibited the degradation of Smc5/6 by HBx (Figure 6F), and this result was similar to that obtained with the HepG2-NTCP infection system (Figure S7 in Supplementary Material). Collectively, TRIM14 interacts with the C-terminal of HBx and might block the role of HBx in promoting HBV replication by rescuing the HBx-mediated degradation of Smc5/6.

### TRIM14 SPRY Domain Is Responsible for HBx Interaction and Inhibition of HBV Replication

**Figure 7:**
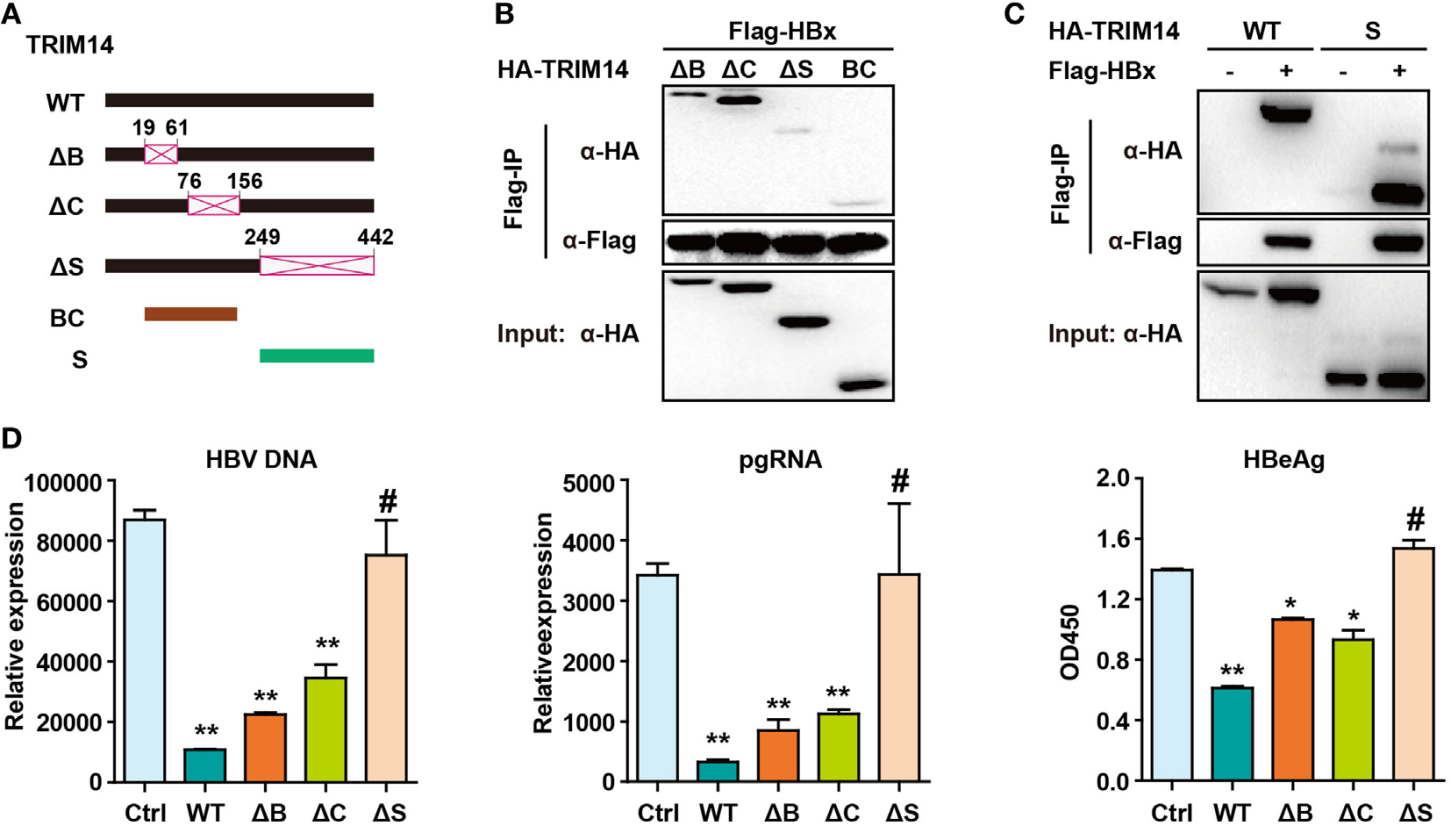
Hepatitis B virus X (HBx) protein interacts with the SPRY domain of TRIM14. **(A)** Schematic illustrations of the mutant *TRIM14* constructs, wherein the numbers indicate the amino acids in the TRIM14 constructs (B, BBOX domain; C, coil domain; S, SPRY domain). **(B)** 293 T cells were transfected with HA-TRIM14 mutants with Flag-tagged HBx expression plasmids as indicated; 40 h later, cells were collected and subjected to co-immunoprecipitation assay; the blot was immunoblotted with antibodies as indicated. **(C)** 293 T cells were co-transfected with Flag-HBx and WT HA-TRIM14 protein-expression plasmids or SPRY as indicated and analyzed as in **(B)**. **(D)** HepG2 cells were transfected with pHBV1.3 plasmids, with or without TRIM14 WT or mutants as indicated; at 72 h, RNA and HBV DNA were isolated from the cells or supernatants and analyzed by quantitative real-time PCR. HBeAg level in the supernatant was determined by ELISA. Data are presented as mean ± SD from three independent experiments. The student’s *t-*test was applied to analyze results. *p < 0.05 and **p < 0.01.

The TRIM14 SPRY domain was reported to target the HCV NS5A protein (36). To identify the particular domain of TRIM14 that is critical for HBx interaction, mutants were generated (Figure 7A) and a co-immunoprecipitation (Co-IP) assay was conducted; deletion of BOX or CC domain showed no effect on the HBx–TRIM14 interaction, and there was no interaction between HBx and BC, which included both the BBOX and CC domains. It was only after the SPRY domain deletion that the interaction of HBx and TRIM14 disappeared (Figure 7B); this was further confirmed by the strong interaction of HBx and the SPRY domain (Figure 7C). We further confirmed that it was only after the deletion of SPRY domain that TRIM14 showed no inhibitory effect on HBV replication (Figure 7D). These data suggest the critical role of the TRIM14 SPRY domain in the interaction with HBx and the inhibition of HBV replication.

## Discussion

In HBV patients, IFN treatment remains an important clinical therapeutic option; however, due to the associated adverse effects and low efficiency, IFN-α is not widely used in clinical settings (37). Therefore, characterization of the underlying mechanism of IFN-α-mediated inhibition of HBV replication is a key imperative. In this study, we identified TRIM14—a molecule that appears to play a key role in the regulation of IFN signaling—as a STAT1-dependent ISG. STAT1 directly binds to the TRIM14 promoter, which is responsible for IFN-α-induced TRIM14 induction. The TRIM14 SPRY domain interacts with the C-terminal of the HBx protein, and this interaction might inhibit the role of HBx in HBV replication.

IFN-Is (IFN-α, -β, -ε, and others) have been recognized as the major antiviral cytokines in vertebrates (38), and constitute one of the most important innate immune responses to viral infections, including HBV infection (39). IFN-I signaling is mediated through the heterodimeric transmembrane receptor composed of IFNAR1 and IFNAR2; crosslinking of IFNAR1/2 results in the activation of JAK1 and tyrosine kinase 2, which further induces phosphorylation of members of the STAT family, thereby facilitating their dimerization and nuclear translocation (40). This results in the subsequent induction of approximately 300 ISGs, which can inhibit different stages of the viral life cycle. Rapid and robust induction of IFN-I is a critical event in the host innate immune antiviral response, and the antiviral function of IFN-I is effected *via* the induction of these approximately 300 downstream ISGs. TRIM14 was shown to regulate IFN production and play an important role in the innate immune response (22). In the present study, we found that TRIM14 was induced by IFN-I in a STAT1-dependent (but not STAT3-dependent) manner, and that STAT1 directly bound to the TRIM14 promoter which activated the transcription of TRIM14. Our results clearly demonstrate the mechanism of TRIM14 production after IFN-I stimulation. We had earlier reported that IL-27 plays an important role in IFN-I-induced upregulation of TRIM25 (21); however, it is interesting to note that the induction of TRIM14 by IFN-I is not IL-27-dependent. Nonetheless, IL-27 was shown to activate STAT1 (21), which in turn promotes the production of TRIM14.

As a positive regulator of IFN-I signaling, TRIM14 may inhibit HBV replication *via* amplification of IFN-I signaling (22). Thus, it is easy to understand the significant inhibitory effect of TRIM14 on HBV replication in pHBV1.2ΔX-transfected HepG2 cells. Here, we identified TRIM14 as an IFN-I-inducible gene and showed that STAT1 (but not STAT3) was essential for the production of TRIM14. STAT1 promotes TRIM14 expression by directly binding to the promoter region of TRIM14, and overexpression of TRIM14 inhibits HBV replication. In Figure 3C, the induction of TRIM14 by polyI:C is very weak; this is likely attributable to the different induction kinetics in response to different stimuli. As shown in Figure 1C, TRIM14 induction was quicker in response to polydAdT. In addition, we found that TRIM14 played an important role in IFN-α-mediated inhibition of HBV; however, IFN-α still inhibited HBV replication in TRIM14 KO cells by inducing some other ISGs that may restrict HBV (Figure S2 in Supplementary Material). Interestingly, HBV replication in IFNAR1 KO HepG2 cells was subdued as compared to that in WT cells (Figure S3 in Supplementary Material). Further investigations are required to explore the exact mechanism.

Some ISGs have already been shown to inhibit HBV replication. For example, *APOBEC3A/B* was shown to play a critical role in the degradation of nuclear HBV cccDNA (41), and *TRIM22* was shown to confer antiviral immunity against HBV *via* its inhibitory effect on viral core promoter activity (42). TRIM14 was reported to inhibit HCV replication (43); however, there are only a few reports about the function of TRIM14 in HBV replication. In this study, we evaluated the hypothesis that TRIM14 is a STAT1-dependent ISG and that overexpression of TRIM14 inhibits HBV replication. Our results indicated that TRIM14 interacts with the C-terminal of the HBx protein, which plays an important role in HBV replication; the C-terminal transactivation domain of HBx was adequate for a stimulation effect on HBV transcription and replication (44), which suggests that the interaction of TRIM14 and the HBx C-terminal may inhibit HBx-mediated activation of HBV gene expression from the cccDNA template (45). Moreover, we found that the inhibitory effect of TRIM14 on HBV replication was much less significant in the pHBV1.2 ΔX plasmid-transfected cells, when compared with that in pHBV1.2 WT plasmid-transfected cells (Figure 3A); this indicated that HBx was indeed the target of TRIM14. Several reports have shown that the HBx-DDB1-CUL4-ROC1 E3 ligase complex targets the SMC5/6 complex to enhance HBV gene expression from episomal cccDNA (14, 46). DDB1 is the best-characterized HBx-binding partner (11), and their interaction is essential for HBV replication. SMC5/6 forms the foundation of a multi-subunit DNA-repair complex, and functions as a restriction factor that selectively blocks extrachromosomal DNA transcription (12). Therefore, it is possible that the interaction of TRIM14 and HBx inhibits the formation of the HBx-DDB1–Smc5/6 complex. As expected, we found that TRIM14 interacted with the C-terminal of HBx, which inhibited the formation of Smc-HBx–DDB1 complex. DDB1 was earlier shown to stabilize HBx (47), and we found that the binding of TRIM14 to HBx blocked this function (Figure 6E). In addition, we observed a weak interaction of HBx with HBV core and large S protein (Figure 6A); this suggests that TRIM14 may play multiple roles *via* its interactions with other HBV proteins, and that it may influence the HBV life cycle at multiple stages. Here, we focused on HBx owing to its strong interaction with TRIM14. Our study clearly demonstrates that TRIM14 is a STAT1-dependent ISG, and that the IFN-I–TRIM14–HBx axis may explain the mechanism of IFN-induced inhibition of viral replication. Nevertheless, more research is needed to further clarify the mechanism underlying the TRIM14–HBx interaction and the role of TRIM14 in HBV infection and replication.

**Figure 8:**
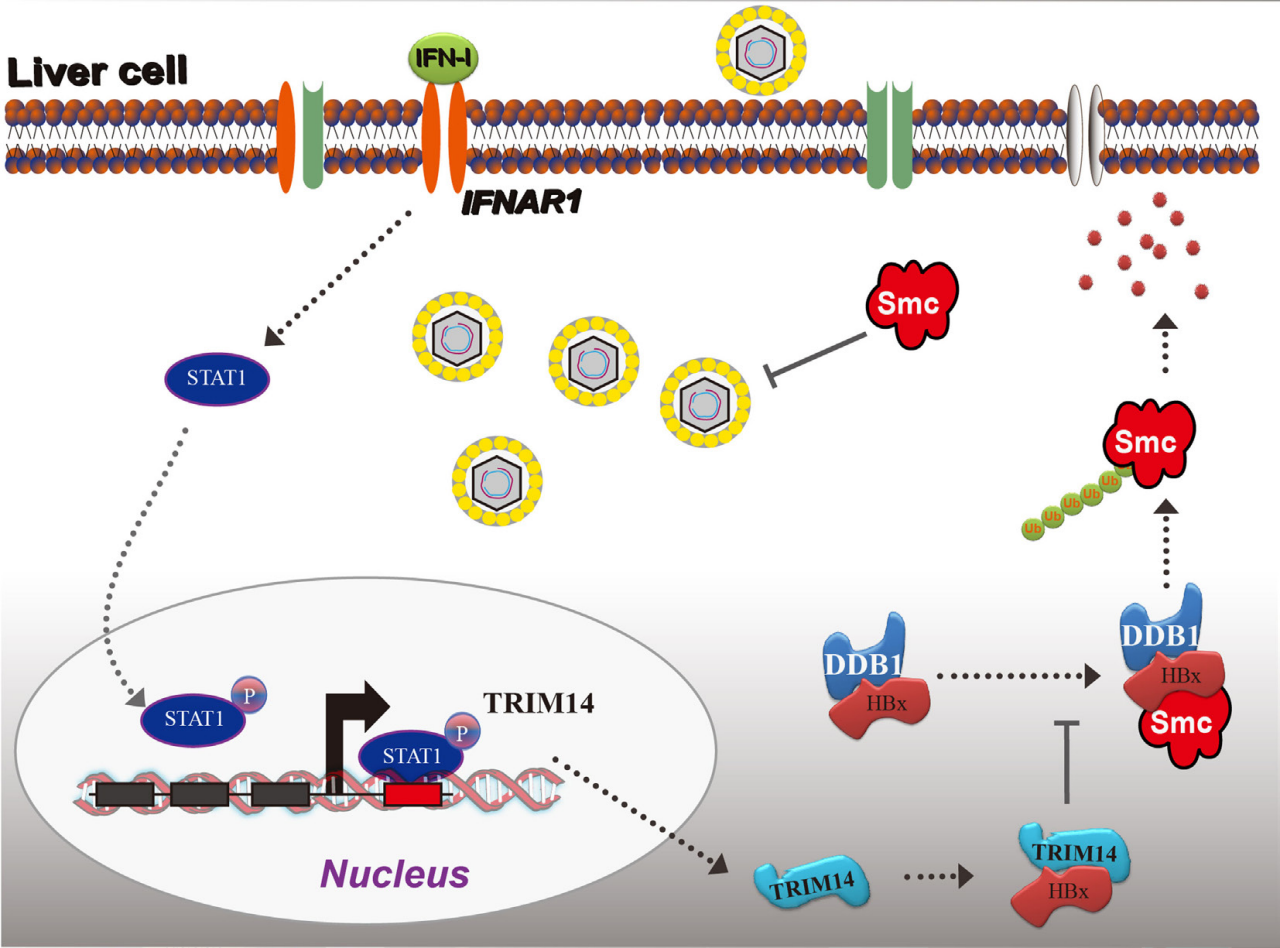
Model depicting TRIM14 as an IFN-I-stimulated gene which controls the replication of hepatitis B virus.

Based on the findings from this study, we propose a working model for IFN-I-induced TRIM14 inhibition of HBV replication by interacting with HBx (Figure 8).

## Conclusion

TRIM14 is an ISG, and the direct binding of STAT1 to the TRIM14 promoter is responsible for IFN-I-mediated TRIM14 induction. TRIM14 interacts with the C-terminal of HBx, and this interaction may inhibit HBx activity by inhibiting the formation of the Smc-HBx–DDB1 complex. Thus, an increase in TRIM14 expression may benefit HBV treatment.

## Ethics Statement

In this study, we enrolled 16 HBV-infected patients who were treated with IFN-α (Table S1 in Supplementary Material). Venous blood sampling was done to collect serum and peripheral blood mononuclear cells (PBMCs) 15 min before or 24, 48, and 96 h after IFN-α treatment. We have only collected 10 patients for the 24 h time-point and 6 patients for 96 h for some reasons. This study (ClinicalTrials.gov number: NCT01671787) was approved by the Ethics Committee of the First Hospital of Jilin University. All subjects signed an informed consent prior to enrolment.

## Author Contributions

GT planned, designed, and performed the experiments and wrote the paper. FX and HS performed the experiments. YY collected the patient’s samples. FQ and FM provided technical assistance and facility. GC gave suggestions and revised the manuscript.

## Conflict of Interest Statement

The authors declare that the research was conducted in the absence of any commercial or financial relationships that could be construed as a potential conflict of interest.

## Supplementary Material

Figure S1 HepG2 cells were transfected with IFN β-Luc together with pGL4.7 TK-Luc reporter. After 8 h, the cells were transfected with polyIC, polydAdT, or pHBV1.3 plasmids and luciferase activity was quantified at 16 h after transfection. The student’s *t-*test was applied to analyze results. ***p* < 0.01 and ^#^*p* > 0.05.

Figure S2 HepG2 cells were treated with IFN-α for 24 h or transfected with polyI:C or polydAdT; 24 h later, a chromatin immunoprecipitation assay was performed to analyze STAT1 binding to sites within the TRIM14 promoter region (site #1 and #2).

Figure S3 **(A)** HepG2 cells were transfected with pHBV1.3 or co-transfected with pHBV1.3 and TRIM14, or left untransfected, as indicated. At 48 h, cells were collected and whole-cell lysates were immunoblotted with HBs, HBc, HA, and tubulin antibodies. **(B)** HBs expression in cells as in **(A)** was analyzed by Immunofluorescence.

Figure S4 HepG2 WT or TRIM14 KO cells were treated with IFN-α for 8 h as indicated and mRNA expressions of IFIT2, IFIT3, and IFIT5 determined by quantitative real-time PCR. Data are presented as mean ± SD from three independent experiments. The student’s *t-*test was applied to analyze results. **p* < 0.05 and ****p* < 0.001.

Figure S5 **(A–C)** HepG2 WT or IFNAR1 KO cells were transfected with pHBV1.3 plasmids, with or without TRIM14, as indicated; at 48 h, RNA and hepatitis B virus DNA were isolated from the cells or the supernatant and analyzed by quantitative real-time PCR. HBeAg level in the supernatant was determined by ELISA. **(D)** Whole-cell lysates were immunoblotted with IFNAR1, HA, or GAPDH antibodies. Data are presented as mean ± SD from three independent experiments. The Student’s *t-*test was applied to analyze results. ***p* < 0.01; ****p* < 0.0001.

Figure S6 HepG2 cells were transfected with GST-HBx plasmids, together with Flag-EV or Flag-TRIM14, as indicated; 48 h later, cells were fixed in acetone-methanol and subjected to immunofluorescence analysis.

Figure S7 HepG2-NTCP cells were transfected with HA-TRIM14 or empty plasmids; 24 h later, cells were infected with hepatitis B virus as indicated. After 7 days, HBsAg in the supernatant was analyzed by ELISA and whole cell lysates were immunoblotted with antibodies as indicated. Data are presented as mean ± SD from three independent experiments. The student’s *t-*test was applied to analyze results. ***p* < 0.01 and **p* < 0.05.
