# Supplementary figures and images for: Identification of TRIM14 as a Type I IFN-Stimulated Gene Controlling Hepatitis B Virus Replication by Targeting HBx
Source: Front Immunol. 2018 Aug 13;9:1872. doi: 10.3389/fimmu.2018.01872 (PMC6100580; doi:10.3389/fimmu.2018.01872)

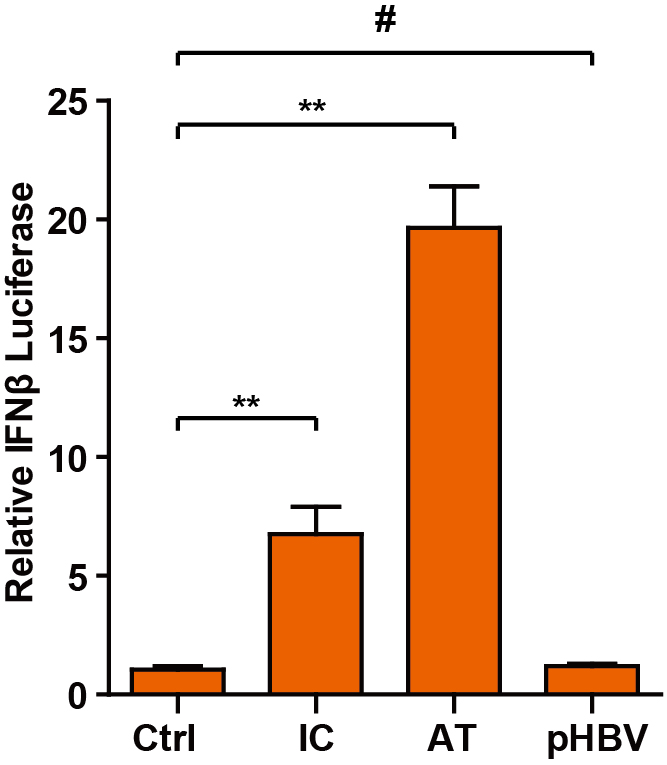

Supplement: Figure S1 — HepG2 cells were transfected with IFN β-Luc together with pGL4.7 TK-Luc reporter. After 8 h, the cells were transfected with polyIC, polydAdT, or pHBV1.3 plasmids and luciferase activity was quantified at 16 h after transfection. The student’s t-test was applied to analyze results. **p < 0.01 and #p > 0.05. [file image_1.jpeg]

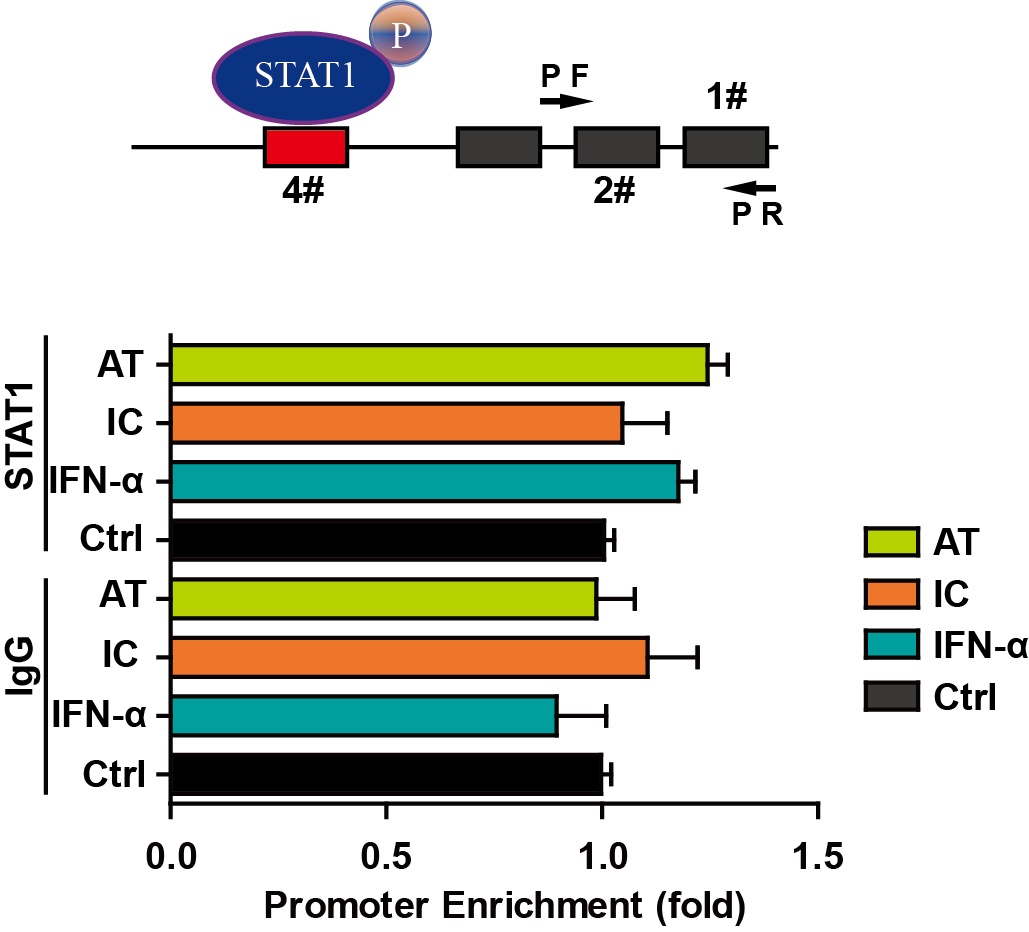

Supplement: Figure S2 — HepG2 cells were treated with IFN-α for 24 h or transfected with polyI:C or polydAdT; 24 h later, a chromatin immunoprecipitation assay was performed to analyze STAT1 binding to sites within the TRIM14 promoter region (site #1 and #2). [file image_2.jpeg]

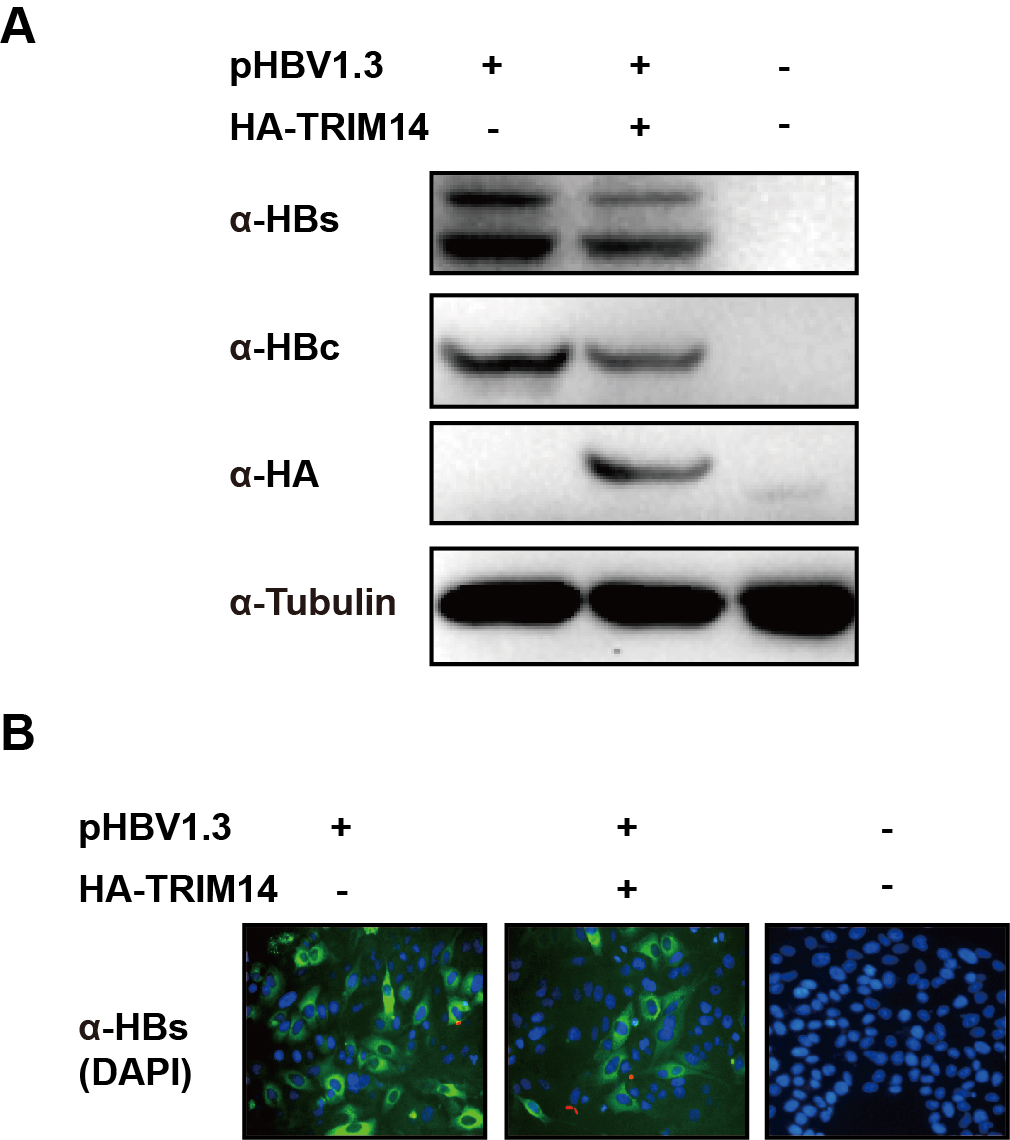

Supplement: Figure S3 — (A) HepG2 cells were transfected with pHBV1.3 or co-transfected with pHBV1.3 and TRIM14, or left untransfected, as indicated. At 48 h, cells were collected and whole-cell lysates were immunoblotted with HBs, HBc, HA, and tubulin antibodies. (B) HBs expression in cells as in (A) was analyzed by Immunofluorescence. [file image_3.jpeg]

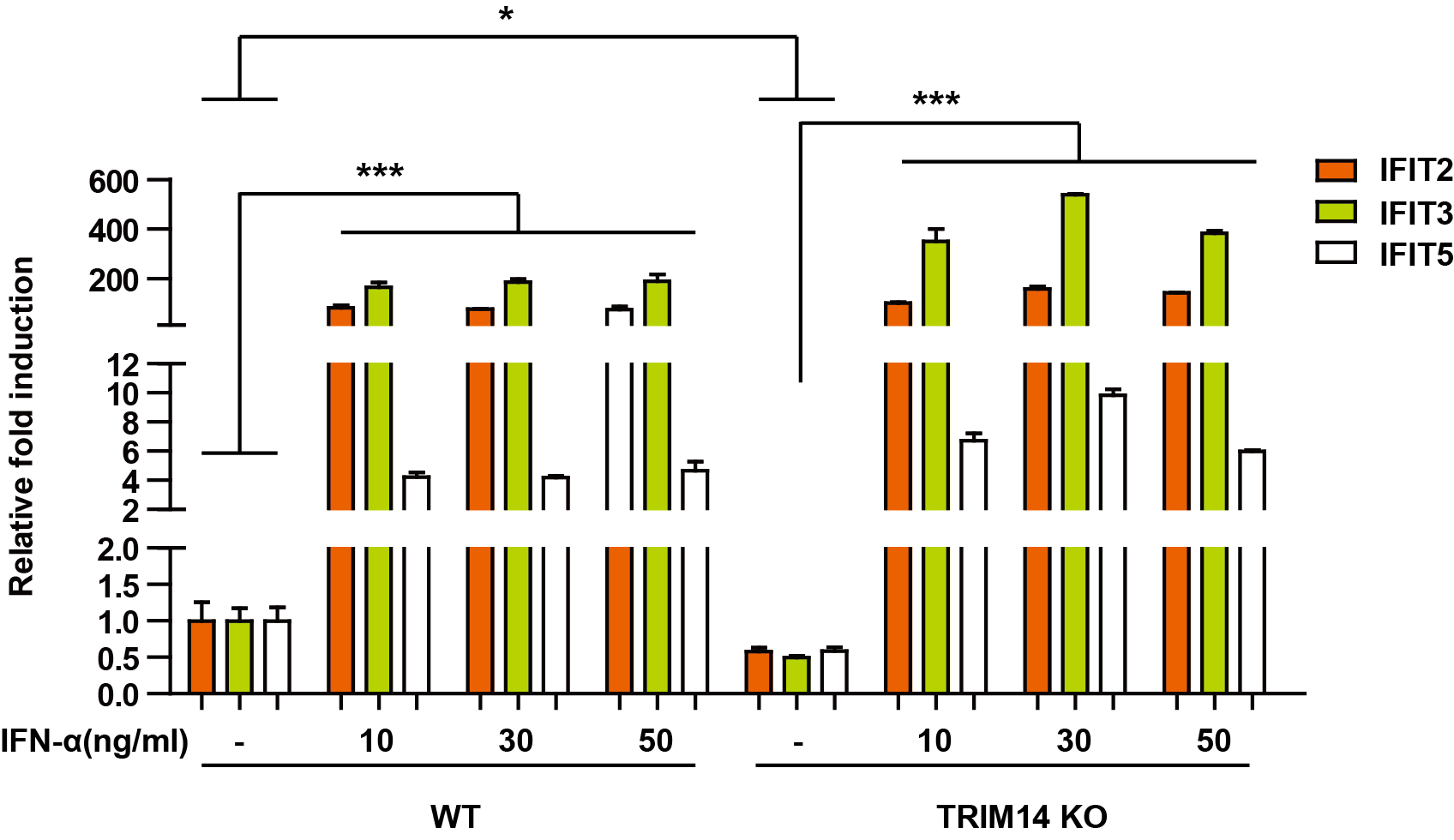

Supplement: Figure S4 — HepG2 WT or TRIM14 KO cells were treated with IFN-α for 8 h as indicated and mRNA expressions of IFIT2, IFIT3, and IFIT5 determined by quantitative real-time PCR. Data are presented as mean ± SD from three independent experiments. The student’s t-test was applied to analyze results. *p < 0.05 and ***p < 0.001. [file image_4.jpeg]

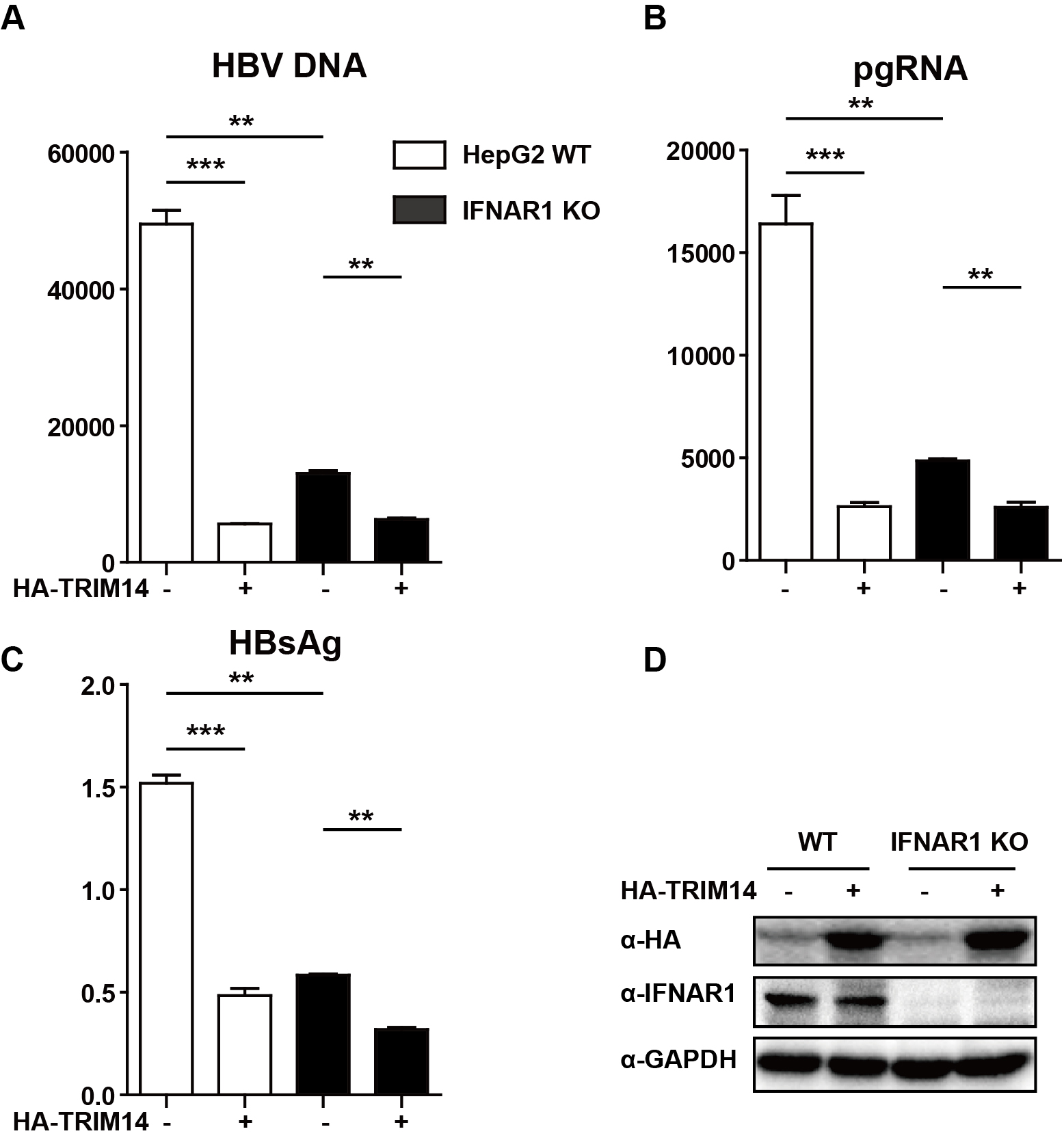

Supplement: Figure S5 — (A–C) HepG2 WT or IFNAR1 KO cells were transfected with pHBV1.3 plasmids, with or without TRIM14, as indicated; at 48 h, RNA and hepatitis B virus DNA were isolated from the cells or the supernatant and analyzed by quantitative real-time PCR. HBeAg level in the supernatant was determined by ELISA. (D) Whole-cell lysates were immunoblotted with IFNAR1, HA, or GAPDH antibodies. Data are presented as mean ± SD from three independent experiments. The Student’s t-test was applied to analyze results. **p < 0.01; ***p < 0.0001. [file image_5.jpeg]

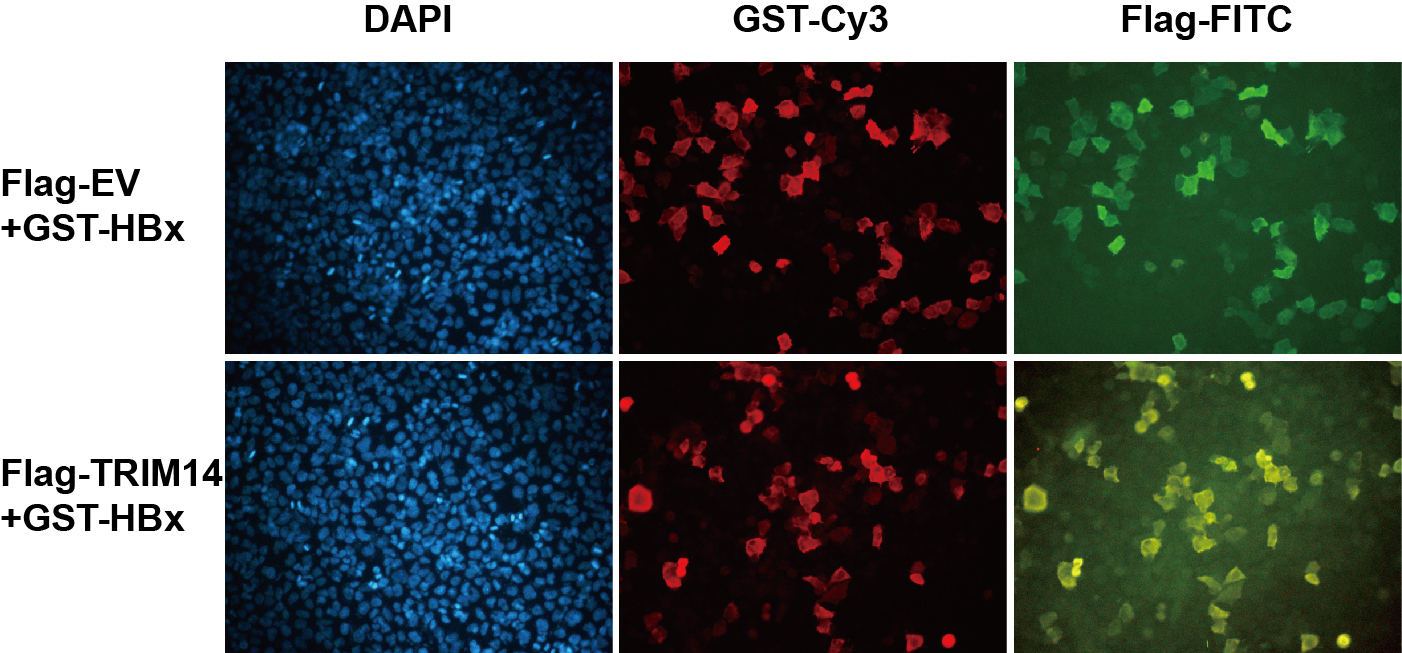

Supplement: Figure S6 — HepG2 cells were transfected with GST-HBx plasmids, together with Flag-EV or Flag-TRIM14, as indicated; 48 h later, cells were fixed in acetone-methanol and subjected to immunofluorescence analysis. [file image_6.jpeg]

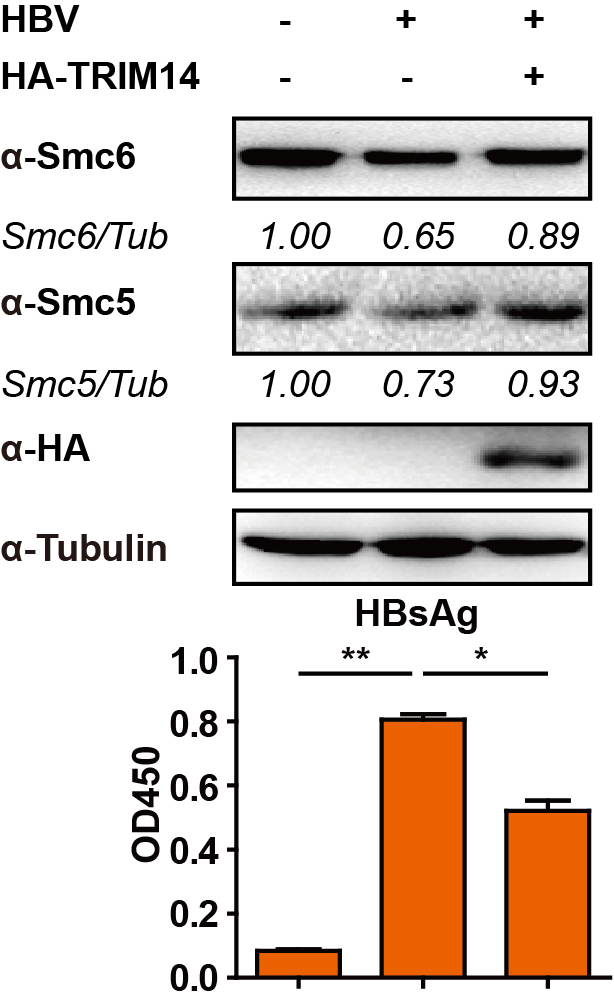

Supplement: Figure S7 — HepG2-NTCP cells were transfected with HA-TRIM14 or empty plasmids; 24 h later, cells were infected with hepatitis B virus as indicated. After 7 days, HBsAg in the supernatant was analyzed by ELISA and whole cell lysates were immunoblotted with antibodies as indicated. Data are presented as mean ± SD from three independent experiments. The student’s t-test was applied to analyze results. **p < 0.01 and *p < 0.05. [file image_7.jpeg]

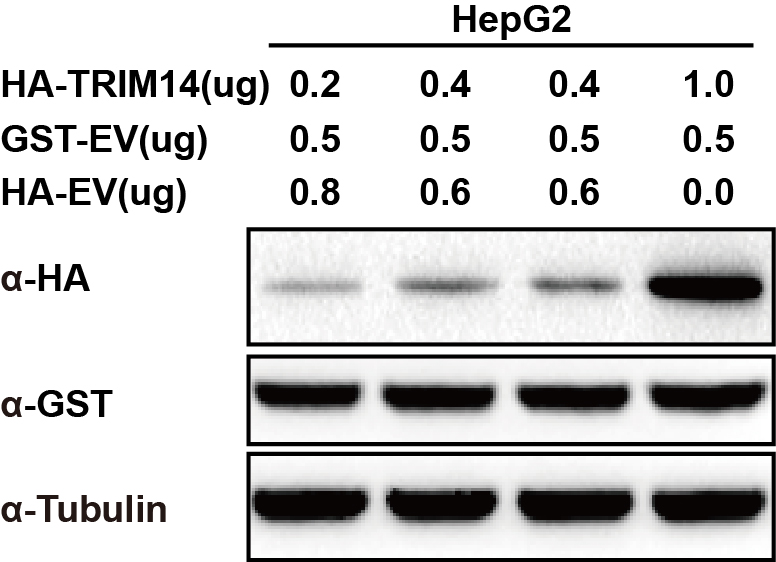

Supplement: Supplementary file 8 [file image_8.jpeg]
