# Supplementary material for: Identification of TRIM14 as a Type I IFN-Stimulated Gene Controlling Hepatitis B Virus Replication by Targeting HBx
Source: Front Immunol. 2018 Aug 13;9:1872. doi: 10.3389/fimmu.2018.01872 (PMC6100580; doi:10.3389/fimmu.2018.01872)
Supplement: Supplementary file 9 [file table_1.doc]

**Table S1. HBV infected patients with IFN treatment**

|  | **before treatment** | | | | **17 weeks after treatment** | | | |
| --- | --- | --- | --- | --- | --- | --- | --- | --- |
| NO | AST0 | ALT0 | HBeAG 0 | HBV DNA 0 | AST 17 | ALT 17 | HBeAG 17 | HBV DNA 17 |
| 1 | 188 | 280 | 963.762 | 170000000 | 63 | 64 | 13.754 | 119000 |
| 2 | 60 | 106 | 861.489 | 170000000 | 59 | 96 | 782.74 | 170000000 |
| 3 | 130 | 162 | 903.773 | 124000000 | 104 | 141 | 657.992 | 14500000 |
| 5 | 78 | 152 | 1368.318 | 170000000 | 21 | 17 | 35.267 | 165000 |
| 6 | 111 | 293 | 1240 | 170000000 | 55 | 117 | 667.605 | 170000000 |
| 7 | 95 | 219 | 675.52 | 170000000 | 73 | 135 | 434.09 | 170000000 |
| 8 | 54 | 108 | 0.455 | 2560000 | 44 | 65 | 0.349 | 296 |
| 9 | 89 | 145 | 1028.726 | 170000000 | 80 | 82 | 20.289 | 1310000 |
| 10 | 89 | 91 | 0.433 | 25400000 | 193 | 273 | 0.415 | 224 |
| 11 | 86 | 227 | 12.351 | 498000 | 39 | 68 | 4.455 | 1120 |
| 12 | 64 | 188 | 1134.673 | 255000000 | 42 | 73 | 1071.894 | 142000000 |
| 13 | 52 | 89 | 1293.93 | 165000000 | 45 | 54 | 803.594 | 5120000 |
| 14 | 88 | 206 | 1045.839 | 170000000 | 83 | 154 | 686.078 | 56200000 |
| 15 | 62 | 172 | 840.371 | 170000000 | 30 | 36 | 2.837 | 1390 |
| 16 | 52 | 89 | 9.171 | 2080000 | 40 | 51 | 2.771 | 3170 |
