# Supplementary material for: Identification of TRIM14 as a Type I IFN-Stimulated Gene Controlling Hepatitis B Virus Replication by Targeting HBx
Source: Front Immunol. 2018 Aug 13;9:1872. doi: 10.3389/fimmu.2018.01872 (PMC6100580; doi:10.3389/fimmu.2018.01872)
Supplement: Supplementary file 10 [file table_2.doc]

**Table S2. SgRNAs , CHIP, Q-PCR and Cloning primers**

| **GENE** | **FORWARD** | **REVERSR** |
| --- | --- | --- |
| ***Q-PCR primers*** | | |
| GAPDH | CGGATTTGGTCGTATTGGG | TCTCGCTCCTGGAAGATGG |
| HBV DNA | GAGTGTGGATTCGCACTCC | GAGGCGAGGGAGTTCTTCT |
| cccDNA | GTGCCTTCTCATCTGCCGG | GGAAAGAAGTCAGAAGGCAA |
| pgRNA | TCTTGCCTTACTTTTGGAAG | AGTTCTTCTTCTAGGGGACC |
| ***Chip primers*** | | |
| Chip(#4) | GTCATCCTCCTAGGGGTCGG | CGCCATTCATCTCCACCTCC |
| Chip(#1,#2) | AATCCTTCTGCTCAGTCCGA | GACCAAAGTTGGCTGTGACC |
| ***Cloning primers*** | | |
| TRIM14 PROMOTER WT | GCTCGCTAGCCTCGAG ACAGTATATGCAATCCTCC | TCTTGATATCCTCGAG ATCGTCCCCACCTGCACG |
| TRIM14 PROMOTER M1 | TATCTTACAGATTATCTTTCCGGG | AAGATAATCTGTAAGATACCTTCA |
| TRIM14 PROMOTER M2 | ATGATTATCAAGGGCAAGAGAGGA | CTTGCCCTTGATAATCATTCCCAC |
| TRIM14 PROMOTER M3 | TTTCTGGGCAGCGTCATTAAGCAG | AATGACGCTGCCCAGAAAACTGCA |
| TRIM14 PROMOTER M4 | CCCACGGCCCTTGGGAGCCCCGCC | GCTCCCAAGGGCCGTGGGCGGGGC |
| TRIM14 ORF cloning | TCGCGGCCGCTCTAGA  ATGGCGGGCGCGGCGAC | AGGCGCCTGGTCTAGA  CTAGGGCAGCCGGGGGAT |
| ***SgRNAs*** | | |
| TRIM14 sgRNA1 | CACCGATCGTGTCAGGATCCAGCGT | AAACACGCTGGATCCTGACACGATC |
| TRIM14 sgRNA2 | CACCGCATCGTGTCAGGATCCAGCG | AAACCGCTGGATCCTGACACGATGC |
| IL27R sgRNA1 | CACCGTTTCCAGCGGACGCGTCCCC | AAACGGGGACGCGTCCGCTGGAAAC |
| IL27R sgRNA2 | CACCCAGTCAGCCTGCATCTCGCC | AAACGGCGAGATGCAGGCTGACTG |
| IFNAR1 sgRNA1 | CACCGACCCTAGTGCTCGTCGCCG | AAACCGGCGACGAGCACTAGGGTC |
| IFNAR1 sgRNA2 | CACCGAAGCAGCACTACTTACGTCA | AAACTGACGTAAGTAGTGCTGCTTC |
